# Measuring Parkinson's disease over time: The real‐world within‐subject reliability of the MDS‐UPDRS

**DOI:** 10.1002/mds.27790

**Published:** 2019-07-10

**Authors:** Luc J.W. Evers, Jesse H. Krijthe, Marjan J. Meinders, Bastiaan R. Bloem, Tom M. Heskes

**Affiliations:** ^1^ Radboud University Medical Center; Donders Institute for Brain, Cognition and Behaviour; Department of Neurology Nijmegen The Netherlands; ^2^ Radboud University; Institute for Computing and Information Sciences Nijmegen The Netherlands; ^3^ Radboud University Medical Center; Radboud Institute for Health Sciences; Scientific Center for Quality of Healthcare (IQ healthcare) Nijmegen The Netherlands

**Keywords:** disease progression, MDS‐UPDRS, modeling, Parkinson's disease, reliability

## Abstract

**Background:**

An important challenge in Parkinson's disease research is how to measure disease progression, ideally at the individual patient level. The MDS‐UPDRS, a clinical assessment of motor and nonmotor impairments, is widely used in longitudinal studies. However, its ability to assess within‐subject changes is not well known. The objective of this study was to estimate the reliability of the MDS‐UPDRS when used to measure within‐subject changes in disease progression under real‐world conditions.

**Methods:**

Data were obtained from the Parkinson's Progression Markers Initiative cohort and included repeated MDS‐UPDRS measurements from 423 de novo Parkinson's disease patients (median follow‐up: 54 months). Subtotals were calculated for parts I, II, and III (in on and off states). In addition, factor scores were extracted from each part. A linear Gaussian state space model was used to differentiate variance introduced by long‐lasting changes from variance introduced by measurement error and short‐term fluctuations. Based on this, we determined the within‐subject reliability of 1‐year change scores.

**Results:**

Overall, the within‐subject reliability ranged from 0.13 to 0.62. Of the subscales, parts II and III (OFF) demonstrated the highest within‐subject reliability (both 0.50). Of the factor scores, the scores related to gait/posture (0.62), mobility (0.45), and rest tremor (0.43) showed the most consistent behavior.

**Conclusions:**

Our results highlight that MDS‐UPDRS change scores contain a substantial amount of error variance, underscoring the need for more reliable instruments to forward our understanding of the heterogeneity in PD progression. Focusing on gait and rest tremor may be a promising approach for an early Parkinson's disease population. © 2019 The Authors. *Movement Disorders* published by Wiley Periodicals, Inc. on behalf of International Parkinson and Movement Disorder Society.

Parkinson's disease (PD) is a chronic and progressive neurodegenerative disease characterized by a heterogeneous symptomatology involving both motor and nonmotor impairments. There is a range of symptomatic treatments, the efficacy of which have been demonstrated at the group level.^1^ An important area of research is the discovery of novel strategies that may slow down or even halt the neurodegenerative process.^2^ But perhaps the most important development is the need to move toward personalized treatments, tailored to each patient's individual profile and needs. Both developments require more fine‐grained insights in progression of PD, ideally at the level of individual patients, or at least tailored to a set of recognizable clinical profiles. Making individual prognostic predictions remains difficult to date, both because of the heterogenous symptomatology of PD and because we lack objective biomarkers.

Recent longitudinal studies, both observational and experimental, have primarily used the Movement Disorder Society — Unified Parkinson's Disease Rating Scale (MDS‐UPDRS) to quantify disease progression.^3^, ^4^ Introduced by the Movement Disorder Society in 2008 as a revision of the original UPDRS, it was designed as a comprehensive instrument for evaluating both motor and nonmotor impairments and disability in PD.^5^ The extent to which the MDS‐UPDRS, or in fact any instrument, is suitable to quantify disease progression, strongly depends on its reliability, that is, an instrument should show reasonably low measurement error in comparison with expected changes, so that the instrument can give a precise estimate of a patient's true progression rate. Using instruments with a high reliability allows for smaller sample sizes and shorter follow‐up in trials assessing new disease‐modifying therapies.^6^ It is also a key prerequisite to build fine‐grained predictive models.

A few aspects might affect the reliability of the MDS‐UPDRS with respect to monitoring changes over time. First, the observer‐rated items are subject to inter‐ and intrarater variability. Second, an individual assessment can only provide a snapshot of the patient's condition and is therefore prone to reflect short‐term effects that are irrelevant to the overall progression of the disease. This is particularly relevant to the motor function assessment (part III) as a substantial proportion of patients experience motor fluctuations as a consequence of dopaminergic therapy (DT). To control for this, longitudinal studies often apply a washout period prior to the motor assessment. However, this can be burdensome for participants, and the length of the washout period (commonly >6 or >12 hours) is not sufficient to cancel out the long duration response of levodopa and dopamine agonists.^7^ In real‐world applications, short‐term effects may also be introduced by factors such as mood, stress, climate, time of the day, and, specifically for parts I and II, whether the patient or caregiver answered the questions.

Despite these sources of variation, the MDS‐UPDRS is often referred to as a highly valid and reliable instrument, which is largely based on two studies examining its clinimetric properties, one conducted by Goetz et al in 2008 (English version) and the other by Martinez‐Martin et al in 2013 (Spanish version).^5^, ^8^ Only the latter examined test‐retest reliability, showing intraclass coefficients (ICCs) of greater than 0.90 for all subscales. However, in the context of monitoring changes over time, the high ICC values should be interpreted with caution because these values only reflect how well the scale can differentiate *between* patients *at a given point*, that is, it is a ratio between the true variance of *absolute* scores and the total variance of *absolute* scores (consisting of the true variance and variance produced by measurement error).^9^ To quantify the scale's ability to assess changes over time, we need a measure of reliability that incorporates the variance of the *within‐subject changes* instead.

The problem becomes apparent when looking at two subsequent 1‐year change scores of the MDS‐UPDRS in the Parkinson's Progression Markers Initiative (PPMI) cohort (Fig. 1). A clear *negative* correlation can be seen, which means that if a MDS‐UPDRS score increases during 1 period, it is likely to decrease in the subsequent period, and vice versa. This behavior is in essence a reflection of the well‐known “regression toward the mean” phenomenon, indicating that the observed changes include a substantial amount of measurement error and are only partly related to changes in the true disease state. These effects can be quantified using models that assume there is an underlying “true” latent phenotype that evolves over time, of which the instrument provides noisy estimates at different times.

**Figure 1:**
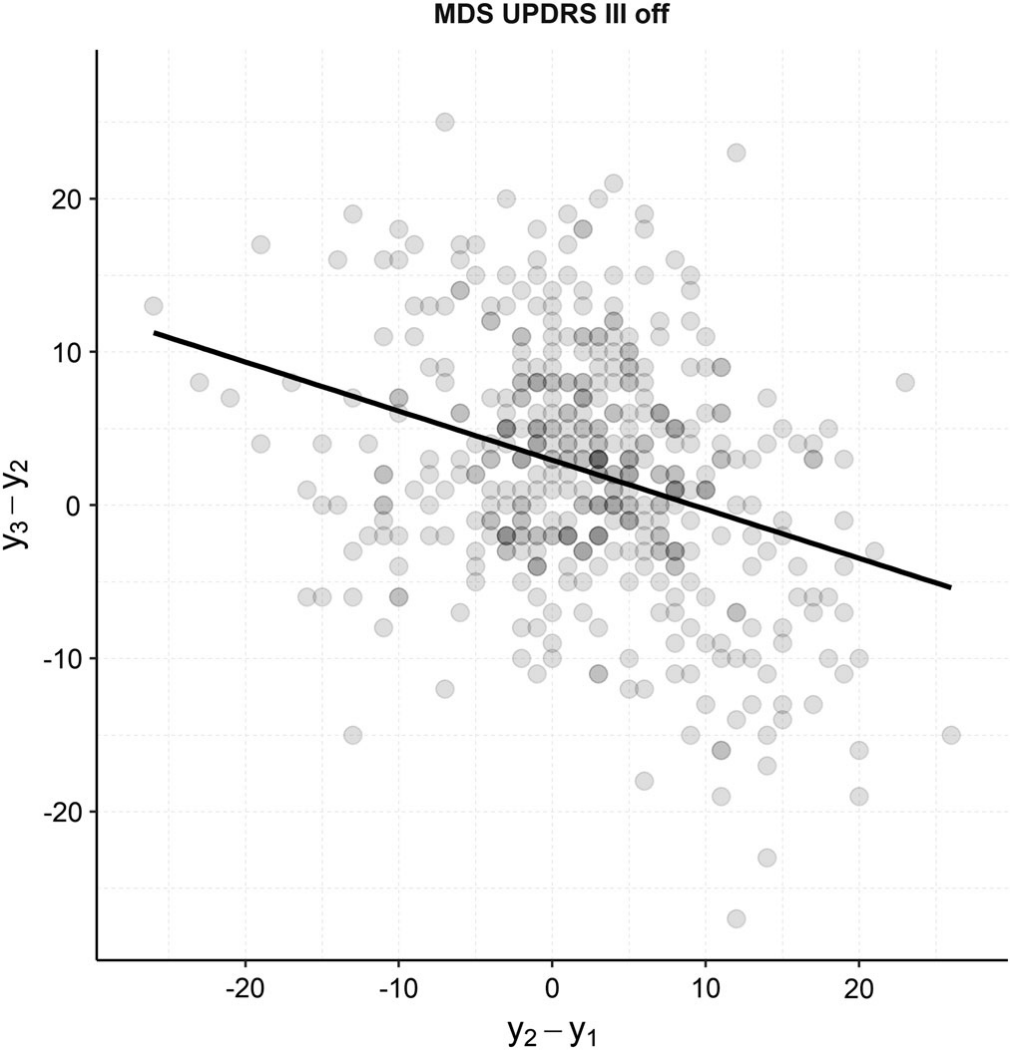
Correlation between two subsequent 1‐year change scores of the MDS‐UPDRS part III (OFF) on the PPMI data set. Line is fitted by linear regression on the response variable y3‐y2. A similar negative correlation can be seen in parts I, II, and III (ON).

## Study Objective

The objective of this study was to use data from a large cohort study to provide a realistic estimate of the reliability of the MDS‐UPDRS when used to measure individual changes in disease progression. By modeling time series of MDS‐UPDRS scores using linear state space models, we aimed to discriminate between variance introduced by actual disease progression and variance introduced by “noise,” consisting of measurement error and short‐term effects irrelevant to the overall disease progression.

## Methods

### PPMI Data Set

The data set used here was obtained from the PPMI database (http://www.ppmi-info.org/data). For up‐to‐date information on the study, please visit http://www.ppmi-info.org. A detailed description of the study design has been published elsewhere.^10^ In brief, PPMI is a multicenter cohort study designed to identify PD progression biomarkers, which was launched in June 2010. Data used here consisted of the MDS‐UPDRS measurements from all included PD subjects and were downloaded from the study website on June 27, 2017.

All sites underwent web‐based and in‐person training in conducting the MDS‐UPDRS.^10^ The complete MDS‐UPDRS was administered at 3‐month intervals during the first year of participation and every 6 months thereafter, up to 5 years after inclusion. We used all available measurements from parts I and II. For part III, we included all annual measurements, because only during these visits did the protocol included scripted OFF state assessments (defined as >6 hours postdose or not receiving any dopaminergic therapy). By also including nonannual visits, we would have introduced bias because these visits only contained OFF assessments from subjects without dopaminergic therapy. It should be noted that the screening visit 1 month before baseline was included, because per protocol no participants were on dopaminergic therapy at inclusion (ie, all assessments could be classified as OFF). At annual visits, part III was repeated approximately 1 hour postdose for subjects receiving dopaminergic therapy. These assessments were classified as ON if performed <6 hours postdose. Part IV was not included in our analysis because it focuses on side effects of dopaminergic therapy and is therefore less suitable for monitoring disease progression.

Subscale scores were computed for parts I, II, and III separately by summing the scores of all containing items.^5^ In addition, we performed a factor analysis separately for parts I, II, and III using all longitudinal MDS‐UPDRS scores that were also included in the other analyses. Factor loadings were computed using the principal component method for parameter estimation, followed by varimax rotation. The decision on the number of factors to retain was determined visually based on the location of the elbow in the scree plot. To compute the factor scores, we summed all items weighted by their factor loadings. The meaning assigned to the identified factors was based on the items with the highest loading. In the analysis, all subscale and factor scores were treated as continuous variables.

### MDS‐UPDRS Progression Model

Observational data sets containing time series can be used to model the measurement error of an instrument using linear state space models.^11^ In this study, a linear Gaussian state space model was used to describe the within‐subject changes in MDS‐UPDRS scores over time, defined by the following 2 equations:

(1) $$y_{t,i}=\theta_{t,i}+v_{t,i}\;v_{t,i}∼N\left(0,,,\sigma_{E}^{2}\right)$$

(2) $$\theta_{t,i}=\theta_{t-1,i}+w_{t,i}\;w_{t,i}∼N\left(\text{trend},\sigma_{\Delta T}^{2}\right)$$

**Figure 2:**
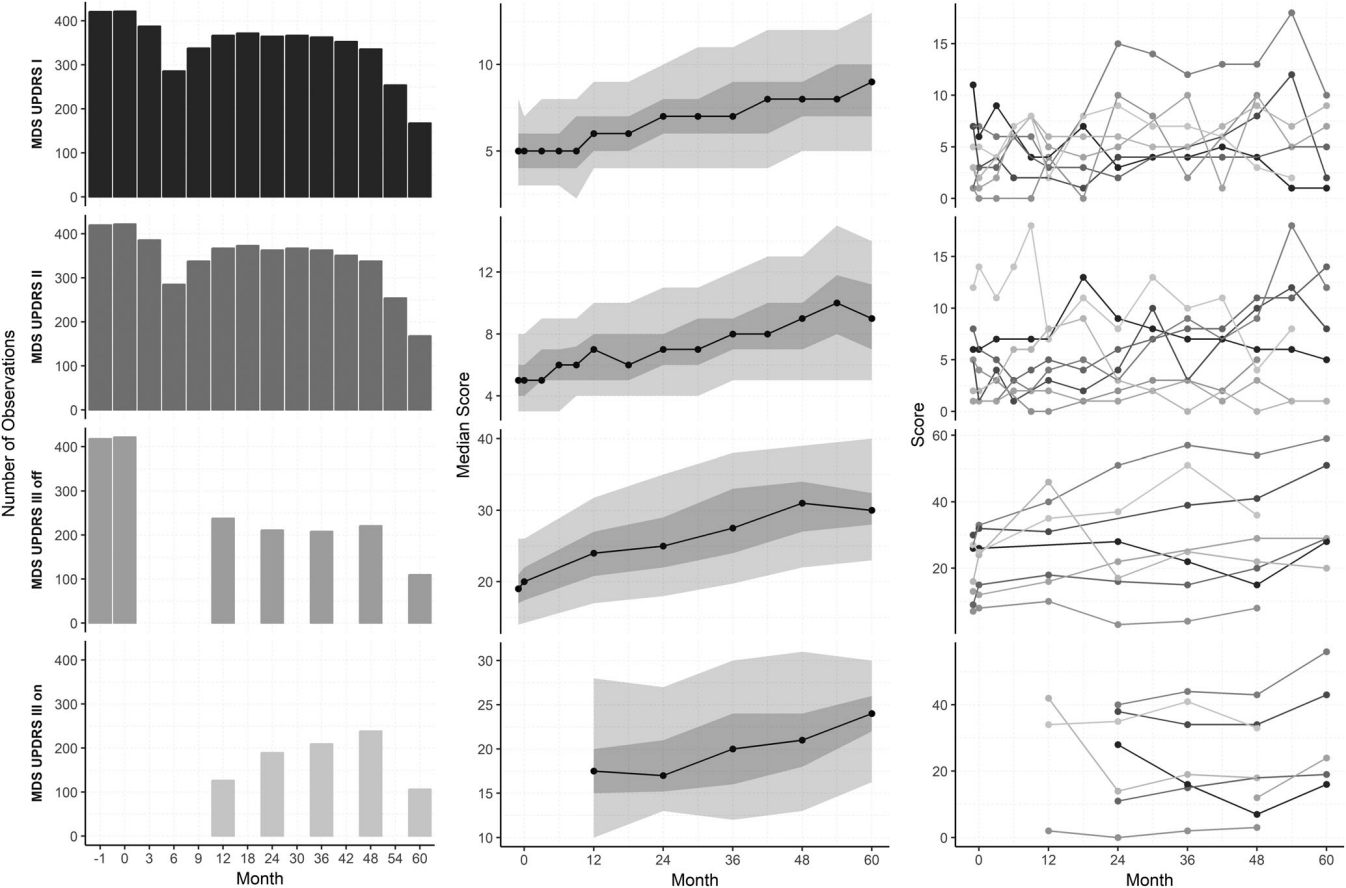
Left: number of assessments included in the analysis for each part and on each visit. Middle: progression of the subscales on a group level (median, 10%, and 25% around the median are shown; missing values were excluded). Right: illustration of the individual progression of the subscale scores (8 illustrative examples of cases with <2 missing values are shown).

**Figure 3:**
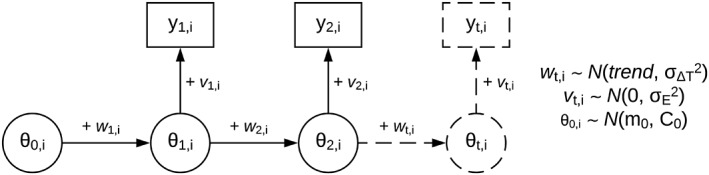
Graphical presentation of the MDS‐UPDRS progression model, as applied to each individual subject denoted by the indices *i*.

Time is indicated by the discrete index *t* which refers to the number of months after screening. Months without measurements in between study visits were treated as missing values. The index *i* refers to an individual subject in the study sample. In equation (1), the observation equation, analogous to classical test theory, we assume that there are hidden true progression states *θ*_*t*,*i*_, which can only be measured indirectly through our observations *y*_*t*,*i*_, the MDS‐UPDRS scores. These observations are the result of the true progression states *θ*_*t*,*i*_ plus Gaussian noise *v*_*t*,*i*_ (independent and identically distributed with mean 0 and variance $\sigma_{E}^{2}$). Thus, in our model $\sigma_{E}^{2}$ is closely related to measurement error, reflecting both inter‐ and intrarater variability and short‐term effects that are irrelevant to disease progression. These effects may be introduced by a wide range of factors such as mood, stress, and climate at the time of the assessment. In equation (2), the state equation, we assume that the true progression states *θ*_*t*,*i*_ from adjacent study visits are linked through Gaussian true progression *w*_*t*,*i*_ (independent and identically distributed with mean *trend* and variance $\sigma_{\Delta T}^{2}$). Therefore, $\sigma_{\Delta T}^{2}$ corresponds to the true variance of change scores. Although factors such as age influence the rate of progression, these were not included in our model because we aimed to estimate the *magnitude* of the variance in true progression $\sigma_{\Delta T}^{2}$, not which factors *explain* this variance. Several Markov assumptions are implied by the model, for instance, given the previous state *θ*_*t* − 1,*i*_ the present state *θ*_*t*,*i*_ is independent of all other past states, and the observed score only depends on the current hidden state. We estimate one *trend* parameter for the study population, independent of the time *t* or individual *i*. This implies that we assume that the average progression in the study population is linear. Given the observed progression of the subscales of the MDS‐UPDRS as displayed in Figure 2 and in the Supplementary Materials, this appears to be a reasonable assumption. Individual progression is not necessarily linear, because *w*_*t*,*i*_ allows for individual variation to the population average *trend*. The specification of the model is completed by defining the initial state *θ*_0,*i*_, which is assumed to be normally distributed with mean *m*_*0*_ and variance *C*_*0*_. These parameters can be estimated without observations at this point using the distribution of the scores at the first point and the assumption of a constant average progression of the study population. See Figure 3 for a graphical representation of the model used in this study.

### Estimation and Validation of Model Parameters

The model parameters were estimated using maximum likelihood estimation, implemented in R using the dlm package dedicated to linear state space models.^12^ For replication purposes, we should note that there is no direct option in the dlm package to add a trend. To achieve this, we introduced a second variable to the hidden states that is set to a constant. So, in our implementation, *θ*_*t*,*i*_ is a vector of length 2, with the first element representing the disease progression state and the second element a constant 1. By properly setting the transition matrix *G* and the variance of *w*_*t*,*i*_, we can keep this variable constant while having the new hidden state depend on the trend (which is a value in *G*): *θ*_*t*,*i*_ = *Gθ*_*t* − 1,*i*_ + *w*_*t*,*i*_. Confidence intervals were calculated using the simple percentile bootstrap method with 1000 repeats: we randomly sampled patients with replacement to construct bootstrap samples and used the 2.5% and 97.5% percentiles of the estimates to obtain 95% confidence intervals. The appropriateness of the model to describe the data was assessed by evaluating the distribution of residuals, that is, the difference between the estimated true progression states and the observed values and the difference between the predicted next state and its measurement.

Based on the estimated parameters, the within‐subject reliability ${\hat{r}}_{\Delta \Delta }$ of 1‐year change scores was calculated as follows:

(3) $$\;{\hat{r}}_{\Delta \Delta }=\frac{{\hat{\sigma }}_{\mathrm{\Delta T}}^{2}}{{\hat{\sigma }}_{\mathrm{\Delta X}}^{2}}=\frac{{\hat{\sigma }}_{\Delta T}^{2}}{{\hat{\sigma }}_{\Delta T}^{2}+2\times {\hat{\sigma }}_{E}^{2}}\;$$

Here, ${\hat{\sigma }}_{\mathrm{\Delta T}}^{2}$ denotes the estimate of the true variance of *change* scores, which is divided by the total variance of 1‐year *change* scores ${\hat{\sigma }}_{\mathrm{\Delta X}}^{2}$, consisting of the true variance plus 2 times the variance produced by measurement error ${\hat{\sigma }}_{E}^{2}$.

In addition, the effect of the length of the levodopa washout period on part III OFF measurements was assessed by comparing the estimates for two different thresholds. Because the median time after DT (for participants receiving DT) was approximately 14 hours, we compared the original threshold (>6 hours postdose) with the threshold >14 hours postdose. To maximize the power of the comparison, we first generated the bootstrap sample before applying the two different thresholds.

## Results

A total of 423 PD subjects were included (277 men and 146 women). At baseline, the average age was 61.7 years, and the average time since diagnosis was 7 months (see Table 1 for all baseline characteristics). The maximum follow‐up period was 60 months. In Figure 2 (left), the number of included assessments can be seen for each visit and for each part of the MDS‐UPDRS. The attrition in the number of included parts I and II assessments was almost completely because of loss to follow‐up. The main reason why fewer part III OFF than parts I and II assessments were included for the annual visits is that not all part III OFF assessments fulfilled the >6 hours postdose criterion. For each part of the MDS‐UPDRS, the progression at the group level and an illustration of individual progression patterns is displayed in Figure 2 (middle and right).

**Table 1:** Baseline characteristics of the study sample (n = 423)

	Mean/percentage	SD	Range
Age (years)	61.7	9.7	33.5–84.8
Sex (% men)	65.5 %	‐	‐
Time since diagnosis of PD (months)	7	0.05	0–36
Hoehn & Yahr (% within group)			
Stage 1	49.2 %	‐	‐
Stage 2	50.8 %	‐	‐
MDS‐UPDRS			
Part I	5.8	4.2	0–22
Part II	5.7	4.2	0–24
Part III (OFF)	20.3	8.9	3–60
Modified Schwab & England	93.9	5.9	70–100
Years of education	15.5	3.0	5–26
Montreal Cognitive Assessment	27.1	2.3	17–30

In part I, three factors were identified (combined explained variance of 46.4%), corresponding to affective symptoms (F1.2), cognitive symptoms (F1.3), and other nonmotor symptoms (F1.1). Also, from part 2 three factors were extracted (combined explained variance of 54%), largely corresponding to impairments in mobility (F2.1), drooling, swallowing and speech (F2.2), and tremor (F2.3). Last, seven factors were identified in part III (with a total explained variance of 62.5%), corresponding to bradykinesia in left extremities (F1), bradykinesia in right extremities (F2), postural instability and gait difficulty (F3), rest tremor (F4), rigidity (F5), facial expression, speech and global bradykinesia (F6), and postural and kinetic tremors (F7); for details see Supplementary Materials. These results are similar but not identical to the factor structure identified by Goetz et al.^5^

### MDS‐UPDRS Progression Model

The estimations of the parameters $\sigma_{E}^{2}$, $\sigma_{\Delta T}^{2}$, trend, and within‐subject reliability, including their 95% confidence interval, are presented in Table 2. Within‐subject reliability varied from 0.13 to 0.62 for the different subscales and factors. Of all subscales, parts II and III (OFF) demonstrated the highest within‐subject reliability. Factor 3.3 (postural instability and gait difficulty), 2.1 (mobility), and 3.4 (rest tremor) were the most reliable factor scores.

**Table 2:** Estimated parameters of the linear state space model, displayed as the estimate on the whole data set and 95% confidence interval as determined by bootstrapping with 1000 samples

	${\hat{\sigma }}_{E}^{2}$	${\hat{\sigma }}_{\mathrm{\Delta T}}^{2}$ a	$\hat{\text{trend}}$ a	${\hat{r}}_{\Delta \Delta }$ a
*Subscales*				
I	4.87 (4.41–5.42)	4.22 (3.35–5.17)	0.92 (0.82–1.02)	0.30 (0.25–0.36)
II	3.66 (3.15–4.19)	7.22 (5.96–8.52)	1.03 (0.91–1.16)	0.50 (0.43–0.56)
III (OFF)	15.52 (12.16–19.24)	31.13 (24.14–38.73)	2.63 (2.34–2.94)	0.50 (0.40–0.60)
III (ON)	27.07 (17.22–35.66)	16.15 (7.44–36.14)	1.04 (0.55–1.48)	0.23 (0.10–0.43)
*Factors part I*				
F1.1 (other nonmotor)	0.29 (0.26–0.32)	0.15 (0.12–0.17)	0.15 (0.13–0.17)	0.20 (0.17–0.24)
F1.2 (affective symptoms)	0.41 (0.35–0.47)	0.16 (0.11–0.21)	0.03 (0.01–0.05)	0.16 (0.11–0.22)
F1.3 (cognitive symptoms)	0.30 (0.25–0.35)	0.26 (0.16–0.39)	0.13 (0.10–0.16)	0.30 (0.20–0.43)
*Factors part II*				
F2.1 (mobility)	0.16 (0.14–0.19)	0.27 (0.22–0.34)	0.17 (0.15–0.20)	0.45 (0.38–0.53)
F2.2 (swallowing, speech)	0.23 (0.20–0.25)	0.15 (0.12–0.19)	0.09 (0.07–0.11)	0.25 (0.21–0.31)
F2.3 (tremor)	0.28 (0.26–0.31)	0.21 (0.17–0.26)	0.02 (0.00–0.04)	0.27 (0.22–0.32)
*Factors part III* (OFF)				
F3.1 (bradykinesia left)	0.14 (0.11–0.16)	0.10 (0.08–0.13)	0.11 (0.09–0.14)	0.27 (0.20–0.35)
F3.2 (bradykinesia right)	0.22 (0.17–0.27)	0.13 (0.08–0.18)	0.11 (0.08–0.13)	0.23 (0.14–0.33)
F3.3 (gait and posture)	0.11 (0.08–0.14)	0.35 (0.19–0.51)	0.10 (0.07–0.14)	0.62 (0.44–0.75)
F3.4 (rest tremor)	0.19 (0.15–0.23)	0.28 (0.20–0.37)	0.10 (0.08–0.13)	0.43 0.33–0.54)
F3.5 (rigidity)	0.26 (0.21–0.30)	0.17 (0.12–0.23)	0.08 (0.05–0.11)	0.25 (0.18–0.34)
F3.6 (other bradykinesia)	0.29 (0.25–0.34)	0.10 (0.07–0.14)	0.05 (0.03–0.08)	0.15 (0.09–0.21)
F3.7 (other tremor)	0.43 (0.36–0.49)	0.13 (0.08–0.19)	‐0.01 (‐0.04 to 0.01)	0.13 (0.08–0.19)

${\hat{\sigma }}_{E}^{2}$ *,* error variance;$\;{\hat{\sigma }}_{\mathrm{\Delta T}}^{2}$ *,* variance in true scores;$\;{\hat{r}}_{\Delta \Delta }$ *,* within‐subject reliability.

a Both ${\hat{\sigma }}_{\mathrm{\Delta T}}^{2}$ and the $\hat{\text{trend}}$ and therefore also ${\hat{r}}_{\Delta \Delta }$ are dependent on the length of the interval. Values here are presented for a follow‐up period of 1 year.

Regarding the part III subscale (OFF), ${\hat{\sigma }}_{E}^{2}$ was significantly lower (difference of –1.75; 95% CI: –2.81 to –0.84), and the within‐person reliability was significantly higher (difference of 0.04; 95% CI: 0.01–0.07) when applying the >14‐hour threshold in comparison with the >6‐hour threshold (*P* < 0.001 based on bootstrap procedure). We should note that because the time since last medication intake was not randomized in this data set, the possibility of confounding should be considered (see Supplementary Materials).

### Model Performance

The QQ plots of parts I and II displaying the model residuals showed heavier tails than the normal distribution, which could be a consequence of both *v*_*t*,*i*_ and/or *w*_*t*,*i*_ having a distribution with heavier tails than the normal distribution (see Supplementary Materials). There were no indications that the data set contained any outliers that might have been caused by erroneous data entry (eg, no values were out of range of feasible MDS‐UPDRS scores), so all data were retained. The distribution of residuals from part III more closely resembled the normal distribution. The inverse correlation between 2 subsequent 1‐year change scores that was presented earlier was similar in data generated based on the model and estimated parameters (see Supplementary Materials).

## Discussion

Our primary aim was to assess the reliability of the MDS‐UPDRS as a tool to measure individual disease progression over time in a population of early‐stage PD patients. A linear Gaussian state space model was applied to a large observational data set to capture the longitudinal behavior of the different subscales and factors. The selected model is closely related to classical test theory, which was the theoretical foundation for previous estimates of the (test‐retest) reliability of the MDS‐UPRS.^9^ The novelty of applying this model to time series of MDS‐UPDRS measurements lies in its ability to provide estimates for both the variance introduced by “noise” (ie, measurement error and short‐term effects) and the variance introduced by long‐lasting differences in individual progression rates. Expanding on previous validation studies, which presented a high *between‐subject* reliability for all parts of the MDS‐UPDRS,^8^ we now demonstrate that the *within‐subject* reliability of all parts is noticeably lower. Parts II and III (OFF) demonstrate a favorable within‐subject reliability compared with parts I and III (ON). The factors related to mobility and tremor demonstrated a relatively consistent behavior and may be, in terms of their reliability, most suitable to monitor individual disease progression in early PD. Last, our findings underscore the importance of considering the symptomatic effects of levodopa when using part III to monitor changes over time, as OFF assessments >6 hours postdose were more consistent than ON assessments, and a longer washout (>14 hours) may further increase the assessment's reliability.

Like any model, ours is a simplification of reality and does not aim to explain the complete behavior of the MDS‐UPDRS, but rather attempts to capture aspects relevant to the question at hand. Still, it is important to explore the assumptions underlying the model and their effect on the relevance of the results. First, some deviations from normality were observed, mostly visible in parts I and II. Although using a distribution with heavier tails might have produced a more accurate fit to the data, it also would have increased the model's complexity, affecting both the complexity of the parameter estimation algorithm and the interpretation of its results.^13^ Given that data sampled from our model and real data display a similar negative correlation between two subsequent change scores, we believe the model is an appropriate choice to capture this remarkable behavior of the MDS‐UPDRS. Second, it was assumed that both $\sigma_{E}^{2}$ and $\sigma_{\Delta T}^{2}$ would remain constant during the course of the disease, which is a simplification; it is reasonable that, for example, the error variance ($\sigma_{E}^{2}$) of part III is larger in populations with a longer disease duration with more severe and unpredictable motor fluctuations. Also, some nonmotor symptoms start to develop later in the disease, which would result in a higher true progression variance ($\sigma_{\Delta T}^{2}$) of part I in this population. Although subjects with de novo PD are often the population of interest in cohort studies on disease progression, the generalizability to other disease stages remains to be evaluated. Given the homogeneity of the PPMI cohort in terms of disease duration at study start and the maximum follow‐up of only 5 years, we believe it was reasonable to estimate one set of variance components that describes the behavior of the MDS‐UPDRS in early PD. This is supported by the observation that the mean and variance of yearly changes did not show any obvious changes over time during the follow‐up period (see Supplementary Materials). Third, the interpretation of the model parameter $\sigma_{\Delta T}^{2}$ deserves some nuance. Although $\sigma_{\Delta T}^{2}$ was referred to as variance in true progression, a more accurate description would be variance in long‐term changes in PD symptomatology. Both the underlying disease progression and long‐term effects of symptomatic treatment (for example, the effect of treatment with antidepressants on part I scores or the gradually increasing dose of dopaminergic medication on parts II and III scores) may contribute to this parameter. Future work may aim to disentangle the contributions from both factors. Last, results presented here are based on one cohort. Although statistical procedures were used to estimate the confidence intervals of the estimations, the results should be validated on independent cohorts.

An important advantage of our approach is that the estimates are based on the actual results from a large multicenter cohort study with all its logistical challenges, in contrast with the highly standardized conditions in which most clinimetric validation studies take place (eg, a small number of participating study centers, a small number of assessors and a short time interval between test and retest, so short‐term effects are more likely to be similar during both). Therefore, our results are more likely to reflect the real‐world behavior of the MDS‐UPDRS when used in this type of study. The presented within‐subject reliabilities can be interpreted as the proportion of variance in MDS‐UPDRS change scores that originate from long‐lasting changes in PD symptomatology and are therefore directly relevant to any cohort study aiming to build predictive models for disease progression. Indeed, the identified behavior of the MDS‐UPDRS may well be an explanation for the results of Latourelle et al, who achieved a higher explained variance (Pearson *R* ^2^) when modeling part III changes in untreated subjects compared with part III (ON) changes in subject receiving DT.^14^ Because error variance is a substantial proportion of variance in all MDS‐UPDRS change scores, the explained variance that can be maximally achieved is limited, and researchers should be aware of the risk of overfitting complex predictive models. It should be noted that it is possible, for example in the case of performing regression on the change scores, to reach a higher explained variance than the presented within‐subject reliabilities by including the baseline score or the previous change score in the model (such as shown in Fig. 1). However, what happens here is that a part of the error variance ($\sigma_{E}^{2}$) is explained in the model, which does not provide any knowledge about the actual disease progression ($\sigma_{\Delta T}^{2}$). The results should also be taken into account when applying (parts of) the MDS‐UPDRS for individual follow‐up in clinical practice, because the literature suggests that changes smaller than the measurement error (*σ*_*E*_), of which we provide estimates, are unlikely to be clinically meaningful.^15^

Although the developers of the MDS‐UPDRS already recommended analyzing the subscales separately instead of analyzing one composite MDS‐UPDRS score, this study allows for a more informed selection of factors and subscales based on their reliability. It deserves some attention that the factor scores related to gait/mobility and rest tremor outperformed the factor scores related to bradykinesia, rigidity, kinetic/postural tremor, and nonmotor symptoms. Initial studies suggested that rest tremor severity measured by the UPDRS did not correlate with disease duration and was therefore not a good marker for progression.^16^, ^17^ However, these studies were all performed in populations with a baseline disease duration of 4–9 years, and later evidence suggested that resting tremor severity does worsen in the early stages of the disease.^18^ Our findings support this and show that, compared with other items (eg, bradykinesia and rigidity), the items related to resting tremor are a relatively reliable way to measure the variation in within‐subject changes of symptom severity in early PD. Although detailed studies on the progression of gait and balance impairments in early PD are rare, Galna et al showed that in this population a deterioration in gait impairment can be observed within 18 months of follow‐up.^19^ In addition, there are indications that early changes in gait/mobility are measurable using the Timed Up & Go test.^20^ We also observed significant progression in gait/posture‐related items of the MDS‐UPDRS in early PD and demonstrated a relatively consistent behavior of these items over time. The latter may be partially explained by the contribution from nondopaminergic pathology, which renders these items less sensitive to the short‐term effects of DT.^18^

In conclusion, our results support the search for more reliable instruments to monitor individual changes in PD symptomatology. In this light, wearable sensors have the potential to overcome some limitations of the MDS‐UPDRS by collecting rater‐independent and continuous data in the patient's own natural environment.^21^ The finding that gait/mobility and tremor‐related items demonstrate the highest within‐subject reliability, highlights the potential of sensor‐based outcome measures in these domains.^22^, ^23^, ^24^, ^25^ Hopefully, the combination of optimally using current clinical rating scales and the development of new reliable instruments will lead to a better understanding of the large heterogeneity in PD progression and pave the way for reliable measures of new disease‐modifying treatments.

## Authors’ Roles

Luc J.W. Evers: conception of research idea, review and critique regarding statistical analysis, writing first draft.

Jesse H. Krijthe: design and execution of statistical analysis, detailed review of first draft.

Marjan J. Meinders: conception and organization of research project, review of manuscript.

Bastiaan R. Bloem: conception of research project, review of manuscript.

Tom M. Heskes: design, review and critique regarding statistical analysis, review of manuscript.

## Financial Disclosures

Luc J.W. Evers is funded by Topconsortium voor Kennis en Innovatie (TKI) Life Sciences & Health (TKI‐LSH), Michael J. Fox Foundation, Philips Research and ZonMW (91215076 “Big Data for Personalised Medicine”). Dr. Jesse H. Krijthe is funded by ZonMw (91215076 “Big Data for Personalised Medicine”). Dr. Marjan J. Meinders has received research support from the Michael J. Fox Foundation, the Stichting Parkinson Fonds, Horizon 2020, and the Topsector Life Sciences and Health. Professor Bastiaan R Bloem currently serves as Associate Editor for the *Journal of Parkinson's Disease*, serves on the editorial of *Practical Neurology*, has received honoraria from serving on the scientific advisory board for Zambon, AbbVie, Biogen, and UCB, has received fees for speaking at conferences from AbbVie, Zambon, and Bial, and has received research support from the Netherlands Organization for Scientific Research, the Michael J. Fox Foundation, UCB, AbbVie, the Stichting Parkinson Fonds, the Hersenstichting Nederland, the Parkinson's Foundation, Verily Life Sciences, Horizon 2020, the Topsector Life Sciences, and Health, and the Parkinson Vereniging. Professor Tom M. Heskes has received research support from NWO Exact Sciences and the EU FP7 projects TACTICS, OPTIMISTIC, and MATRICS.

## Supporting information


**Appendix S1:** Supporting Information
