# Supplementary material for: Measuring Parkinson's disease over time: The real‐world within‐subject reliability of the MDS‐UPDRS
Source: Mov Disord. 2019 Jul 10;34(10):1480–7. doi: 10.1002/mds.27790 (PMC6851993; doi:10.1002/mds.27790)
Supplement: Supplementary file 1 — Appendix S1: Supporting Information [file MDS-34-1480-s001.docx]

# Supplementary materials

## A: Results PCA and varimax rotation

| item | F1.1 | F1.2 | F1.3 |
| --- | --- | --- | --- |
| 1.8 DAYTIME SLEEPINESS | 0.681 |  |  |
| 1.9 PAIN | 0.655 |  |  |
| 1.13 FATIGUE | 0.616 | 0.363 |  |
| 1.7 SLEEP PROBLEMS | 0.608 |  |  |
| 1.12 LIGHT HEADEDNESS ON STANDING | 0.507 |  |  |
| 1.10 URINARY PROBLEMS | 0.498 |  | 0.421 |
| 1.11 CONSTIPATION PROBLEMS | 0.405 |  | 0.427 |
| 1.3 DEPRESSED MOOD |  | 0.787 |  |
| 1.4 ANXIOUS MOOD |  | 0.742 |  |
| 1.5 APATHY |  | 0.652 |  |
| 1.2 HALLUCINATIONS AND PSYCHOSIS |  |  | 0.753 |
| 1.1 COGNITIVE IMPAIRMENT |  | 0.397 | 0.606 |
| 1.6 DOPAMINE DYSREGULATION SYNDROME |  |  |  |

Table 1: item loadings after applying a varimax rotation on the principal components of MDS-UPDRS part I. All absolute loadings higher than 0.3 are displayed.

|  | F1.1 | F1.2 | F1.3 |
| --- | --- | --- | --- |
| *Eigenvalues* | 2.399 | 2.091 | 1.546 |
| *Factor explained variance* | 0.185 | 0.161 | 0.119 |
| *Cumulative explained variance* | 0.185 | 0.345 | 0.464 |

Table 2: eigenvalues and explained variance of the part I factor scores identified by PCA and varimax rotation.

| **ITEM** | **F2.1** | **F2.2** | **F2.3** |
| --- | --- | --- | --- |
| 2.11 GETTING OUT OF BED | 0.775 |  |  |
| 2.9 TURNING IN BED | 0.737 |  |  |
| 2.12 WALKING AND BALANCE | 0.726 |  |  |
| 2.5 DRESSING | 0.661 | 0.348 |  |
| 2.13 FREEZING | 0.584 |  |  |
| 2.8 DOING HOBBIES | 0.556 | 0.318 | 0.397 |
| 2.6 HYGIENE | 0.531 | 0.323 |  |
| 2.4 EATING TASKS | 0.425 | 0.398 | 0.405 |
| 2.1 SPEECH |  | 0.759 |  |
| 2.2 SALIVA & DROOLING |  | 0.708 |  |
| 2.3 CHEWING AND SWALLOWING |  | 0.595 |  |
| 2.10 TREMOR |  |  | 0.886 |
| 2.7 HANDWRITING |  | 0.453 | 0.457 |

Table 3: item loadings after applying a varimax rotation on the principal components of MDS-UPDRS part II. All absolute loadings higher than 0.3 are displayed.

|  | F2.1 | F2.2 | F2.3 |
| --- | --- | --- | --- |
| *Eigenvalues* | 3.384 | 2.226 | 1.416 |
| *Factor explained variance* | 0.260 | 0.171 | 0.109 |
| *Cumulative explained variance* | 0.260 | 0.432 | 0.540 |

Table 4: eigenvalues and explained variance of the part II factor scores identified by PCA and varimax rotation.

| ITEM | F3.1 | F3.2 | F3.3 | F3.4 | F3.5 | F3.6 | F3.7 |
| --- | --- | --- | --- | --- | --- | --- | --- |
| 3.4 FINGER TAPPING L | 0.815 |  |  |  |  |  |  |
| 3.5 HAND MOVEMENTS L | 0.832 |  |  |  |  |  |  |
| 3.6 PRONATION-SUPINATION L | 0.825 |  |  |  |  |  |  |
| 3.7 TOE TAPPING L | 0.824 |  |  |  |  |  |  |
| 3.8 LEG AGILITY L | 0.757 |  |  |  |  |  |  |
| 3.4 FINGER TAPPING R |  | 0.794 |  |  |  |  |  |
| 3.5 HAND MOVEMENTS R |  | 0.792 |  |  |  |  |  |
| 3.6 PRONATION-SUPINATION R |  | 0.802 |  |  |  |  |  |
| 3.7 TOE TAPPING R |  | 0.757 |  |  |  |  |  |
| 3.8 LEG AGILITY R |  | 0.711 | 0.364 |  |  |  |  |
| 3.11 FREEZING OF GAIT |  |  | 0.627 |  |  |  |  |
| 3.10 GAIT |  |  | 0.594 |  |  | 0.327 |  |
| 3.12 POSTURAL STABILITY |  |  | 0.778 |  |  |  |  |
| 3.13 POSTURE |  |  | 0.504 |  |  | 0.464 |  |
| 3.9 ARISING FROM CHAIR |  |  | 0.755 |  |  |  |  |
| 3.14 BODY BRADYKINESIA | 0.378 |  |  |  |  | 0.570 |  |
| 3.2 FACIAL EXPRESSION |  |  |  |  |  | 0.680 |  |
| 3.1 SPEECH |  |  |  |  |  | 0.607 |  |
| 3.17 REST TREMOR AMPLITUDE Lip/Jaw |  |  |  | 0.476 |  |  |  |
| 3.17 REST TREMOR AMPLITUDE LLE |  |  |  | 0.594 |  |  |  |
| 3.17 REST TREMOR AMPLITUDE LUE | 0.488 |  |  | 0.487 |  |  |  |
| 3.17 REST TREMOR AMPLITUDE RLE |  |  |  | 0.584 |  |  |  |
| 3.17 REST TREMOR AMPLITUDE RUE | -0.406 | 0.319 |  | 0.533 |  |  |  |
| 3.18 CONSTANCY OF REST TREMOR |  |  |  | 0.777 |  |  |  |
| 3.16 KINETIC TREMOR OF THE HANDS L | 0.343 |  |  |  |  |  | 0.702 |
| 3.16 KINETIC TREMOR OF THE HANDS R |  |  |  |  |  |  | 0.727 |
| 3.15 POSTURAL TREMOR L | 0.395 |  |  |  |  |  | 0.609 |
| 3.15 POSTURAL TREMOR R |  |  |  |  |  |  | 0.606 |
| 3.3 RIGIDITY LLE | 0.503 |  |  |  | 0.679 |  |  |
| 3.3 RIGIDITY LUE | 0.646 |  |  |  | 0.482 |  |  |
| 3.3 RIGIDITY Neck | 0.310 |  |  |  | 0.520 | 0.308 |  |
| 3.3 RIGIDITY RUE |  | 0.563 |  |  | 0.538 |  |  |
| 3.3 RIGIDITY RLE |  | 0.392 |  |  | 0.753 |  |  |

Table 5: item loadings after applying a varimax rotation on the principal components of MDS-UPDRS part III (OFF). All absolute loadings higher than 0.3 are displayed.

|  | F3.1 | F3.2 | F3.3 | F3.4 | F3.5 | F3.6 | F3.7 |
| --- | --- | --- | --- | --- | --- | --- | --- |
| *Eigenvalues* | 5.340 | 4.232 | 2.634 | 2.215 | 2.112 | 2.045 | 2.032 |
| *Factor explained variance* | 0.162 | 0.128 | 0.080 | 0.067 | 0.064 | 0.062 | 0.062 |
| *Cumulative explained variance* | 0.162 | 0.290 | 0.370 | 0.437 | 0.501 | 0.563 | 0.625 |

Table 6: eigenvalues and explained variance of the part III (OFF) factor scores identified by PCA and varimax rotation.

## B: All estimated parameters of the linear state space model

|  | ${\hat{\boldsymbol{\sigma}}}_{\boldsymbol{E}}^{\boldsymbol{2}}$ | ${\hat{\boldsymbol{\sigma}}}_{\boldsymbol{\Delta}\boldsymbol{T}}^{\boldsymbol{2}}$* | $\hat{\boldsymbol{trend}}$* | ${\hat{\boldsymbol{m}}}_{\boldsymbol{0}}$ | ${\hat{\boldsymbol{C}}}_{\boldsymbol{0}}$ | $\boldsymbol{r̂}_{\boldsymbol{\Delta\Delta}}$* | Log likelihood |
| --- | --- | --- | --- | --- | --- | --- | --- |
| I | 4.87  (4.41–5.42) | 4.22  (3.35–5.17) | 0.92  (0.82–1.02) | 5.44  (5.09-5.78) | 11.82  (9.48-14.28) | 0.30  (0.25–0.36) | -1.62 |
| II | 3.66  (3.15–4.19) | 7.22  (5.96–8.52) | 1.03  (0.91–1.16) | 5.75  (5.35-6.14) | 12.85  (10.29-15.55) | 0.50  (0.43–0.56) | -1.61 |
| III (OFF) | 15.52  (12.16–19.24) | 31.13  (24.14–38.73) | 2.63  (2.34–2.94) | 20.16  (19.35-20.95) | 57.44  (46.85-68.13) | 0.50  (0.40–0.60) | -2.52 |
| III (ON) | 27.07  (17.22–35.66) | 16.15  (7.44–36.14) | 1.04  (0.55–1.48) | 18.89  (17.19-20.82) | 64.80  (38.98-86.25) | 0.23  (0.10–0.43) | -2.69 |

Table 7: all estimated parameters for the MDS-UPDRS subscales, displayed as the estimate on the whole dataset and 95% confidence interval as determined by bootstrapping with 1000 samples.

$\hat{\sigma}_{E}^{2}$: error variance; $\hat{\sigma}_{\Delta T}^{2}$ variance in true scores; $\hat{m}_{0}$: initial mean; $\hat{C}_{0}$: initial variance; Log Likelihood: average log likelihood; ${r̂}_{\Delta\Delta}$*: within-subject reliability.

*both $\hat{\sigma}_{\Delta T}^{2}$ and the $\hat{trend}$, and therefore also ${r̂}_{\Delta\Delta}$, are dependent on the length of the interval. Values here are presented for a follow-up period of 1 year.


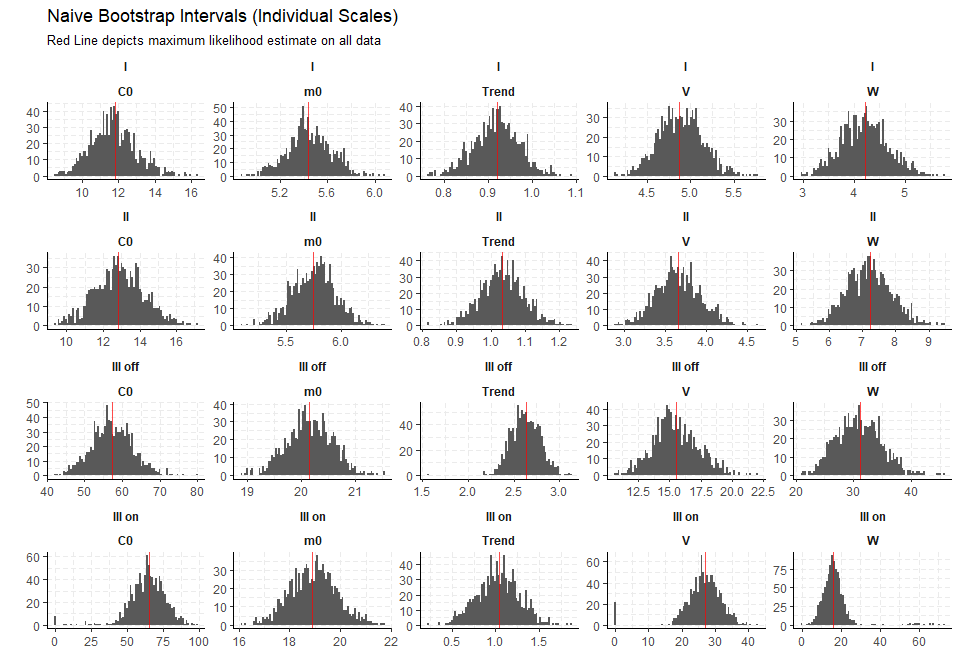


Figure 1: results of the bootstrap procedure with subsequent estimation of all parameters in the linear state space model (red line depicts estimate on whole dataset). Each row presents a different subscale of the MDS-UPDRS. V represents the error variance $\hat{\sigma}_{E}^{2}$, W the variance in true scores $\hat{\sigma}_{\Delta T}^{2}$.

|  | ${\hat{\boldsymbol{\sigma}}}_{\boldsymbol{E}}^{\boldsymbol{2}}$ | ${\hat{\boldsymbol{\sigma}}}_{\boldsymbol{\Delta}\boldsymbol{T}}^{\boldsymbol{2}}$* | $\hat{\boldsymbol{trend}}$* | ${\hat{\boldsymbol{m}}}_{\boldsymbol{0}}$ | ${\hat{\boldsymbol{C}}}_{\boldsymbol{0}}$ | $\boldsymbol{r̂}_{\boldsymbol{\Delta\Delta}}$* | Log likelihood |
| --- | --- | --- | --- | --- | --- | --- | --- |
| ***Part I*** | | | | | | | |
| F1.1 | 0.29  (0.26-0.32) | 0.15  (0.12-0.17) | 0.15  (0.13-0.17) | -0.29  (-0.36- -0.21) | 0.45  (0.37-0.54) | 0.20  (0.17-0.24) | -0.14 |
| F1.2 | 0.41  (0.35-0.47) | 0.16  (0.11-0.21) | 0.03  (0.01-0.05) | -0.05  (-0.11-0.02) | 0.31  (0.23-0.39) | 0.16  (0.11-0.22) | -0.26 |
| F1.3 | 0.30  (0.25-0.35) | 0.26  (0.16-0.39) | 0.13  (0.10-0.16) | -0.27  (-0.32- -0.22) | 0.15  (0.09-0.23) | 0.30  (0.20-0.43) | -0.17 |
| ***Part II*** | | | | | | | |
| F2.1 | 0.16  (0.14-0.19) | 0.27  (0.22-0.34) | 0.17  (0.15-0.20) | -0.33  (-0.39- -0.26) | 0.34  (0.28-0.43) | 0.45  (0.38-0.53) | -0.01 |
| F2.2 | 0.23  (0.20-0.25) | 0.15  (0.12-0.19) | 0.09  (0.07-0.11) | -0.18  (-0.26- -0.11) | 0.51  (0.40-0.62) | 0.25  (0.21-0.31) | -0.06 |
| F2.3 | 0.28  (0.26-0.31) | 0.21  (0.17-0.26) | 0.02  (0.00-0.04) | -0.06  (-0.14-0.01) | 0.41  (0.32-0.51) | 0.27  (0.22-0.32) | -0.16 |
| ***Part III*** | | | | | | | |
| F3.1 | 0.14  (0.11-0.16) | 0.10  (0.08-0.13) | 0.11  (0.09-0.14) | -0.15  (-0.24- -0.07) | 0.76  (0.68-0.85) | 0.27  (0.20-0.35) | -0.04 |
| F3.2 | 0.22  (0.17-0.27) | 0.13  (0.08-0.18) | 0.11  (0.08-0.13) | -0.10  (-0.19- -0.01) | 0.71  (0.64-0.81) | 0.23  (0.14-0.33) | -0.19 |
| F3.3 | 0.11  (0.08-0.14) | 0.35  (0.19-0.51) | 0.10  (0.07-0.14) | -0.23  (-0.28- -0.18) | 0.15  (0.10-0.20) | 0.62  (0.44-0.75) | -0.05 |
| F3.4 | 0.19  (0.15-0.23) | 0.28  (0.20-0.37) | 0.10  (0.08-0.13) | -0.11  (-0.18- -0.05) | 0.41  (0.32-0.50) | 0.43  0.33-0.54) | -0.21 |
| F3.5 | 0.26  (0.21-0.30) | 0.17  (0.12-0.23) | 0.08  (0.05-0.11) | -0.19  (-0.26- -0.11) | 0.50  (0.40-0.61) | 0.25  (0.18-0.34) | -0.25 |
| F3.6 | 0.29  (0.25-0.34) | 0.10  (0.07-0.14) | 0.05  (0.03-0.08) | -0.09  (-0.17- -0.01) | 0.54  (0.45-0.64) | 0.15  (0.09-0.21) | -0.24 |
| F3.7 | 0.43  (0.36-0.49) | 0.13  (0.08-0.19) | -0.01  (-0.04-0.01) | 0.08  (-0.00-0.16) | 0.46  (0.36-0.56) | 0.13  (0.08-0.19) | -0.37 |

Table 8: all estimated parameters for the MDS-UPDRS factors, displayed as the estimate on the whole dataset and 95% confidence interval as determined by bootstrapping with 1000 samples.

$\hat{\sigma}_{E}^{2}$: error variance; $\hat{\sigma}_{\Delta T}^{2}$ variance in true scores; $\hat{m}_{0}$: initial mean; $\hat{C}_{0}$: initial variance; Log Likelihood: average log likelihood; ${r̂}_{\Delta\Delta}$*: within-subject reliability.

*both $\hat{\sigma}_{\Delta T}^{2}$ and the $\hat{trend}$, and therefore also ${r̂}_{\Delta\Delta}$, are dependent on the length of the interval. Values here are presented for a follow-up period of 1 year.

## D: Comparison between the different thresholds for the part III OFF measurements

| ${\hat{\boldsymbol{\sigma}}}_{\boldsymbol{E}}^{\boldsymbol{2}}$ | ${\hat{\boldsymbol{\sigma}}}_{\boldsymbol{\Delta}\boldsymbol{T}}^{\boldsymbol{2}}$* | $\hat{\boldsymbol{trend}}$* | ${\hat{\boldsymbol{m}}}_{\boldsymbol{0}}$ | ${\hat{\boldsymbol{C}}}_{\boldsymbol{0}}$ | $\boldsymbol{r̂}_{\boldsymbol{\Delta\Delta}}$* |
| --- | --- | --- | --- | --- | --- |
| 1.75  (0.84–2.81) | -1.22  (-4.00–1.29) | -0.04  (-0.19–0.10) | 0.01  (-0.04–0.05) | -1.38  (-2.36–-0.35) | -0.04  (-0.07–-0.01) |

Table 9: Estimated difference of the model parameters for MDS-UPDRS part III off measurements between the thresholds >6 hours minus >14 hours since last medication intake, displayed as the median and confidence interval given by the bootstrap procedure (1000 repeats).

$\hat{\sigma}_{E}^{2}$: error variance; $\hat{\sigma}_{\Delta T}^{2}$ variance in true scores; $\hat{m}_{0}$: initial mean; $\hat{C}_{0}$: initial variance; Log Likelihood: average log likelihood; ${r̂}_{\Delta\Delta}$*: within-subject reliability.

*both $\hat{\sigma}_{\Delta T}^{2}$ and the $\hat{trend}$, and therefore also ${r̂}_{\Delta\Delta}$, are dependent on the length of the interval. Values here are presented for a follow-up period of 1 year.


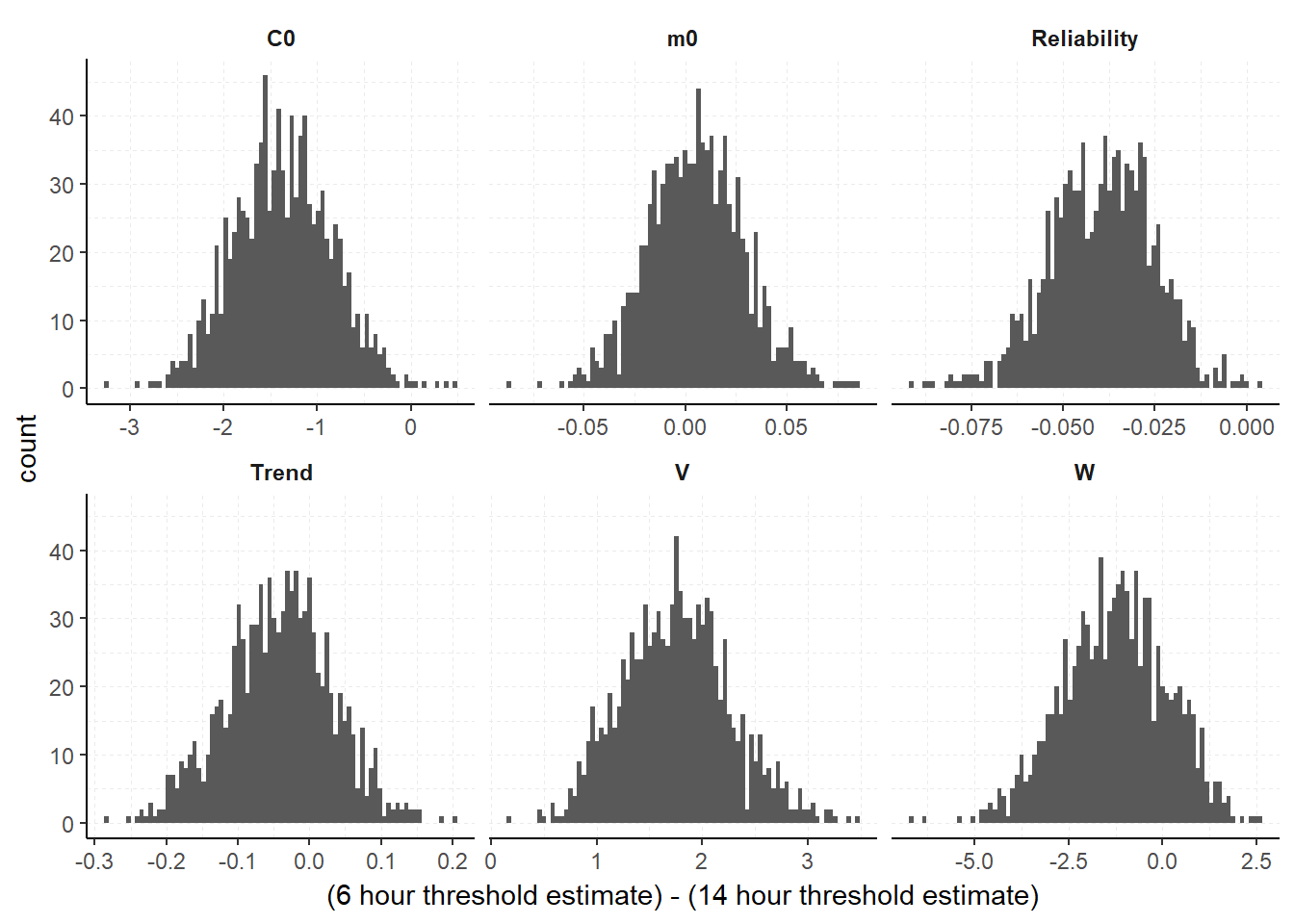


Figure 2: results of the bootstrap procedure with subsequent estimation the difference between thresholds >6 hours minus >14 hours post dose. V represents the error variance $\hat{\sigma}_{E}^{2}$, W the variance in true scores $\hat{\sigma}_{\Delta T}^{2}$.

## E: Distribution of model residuals


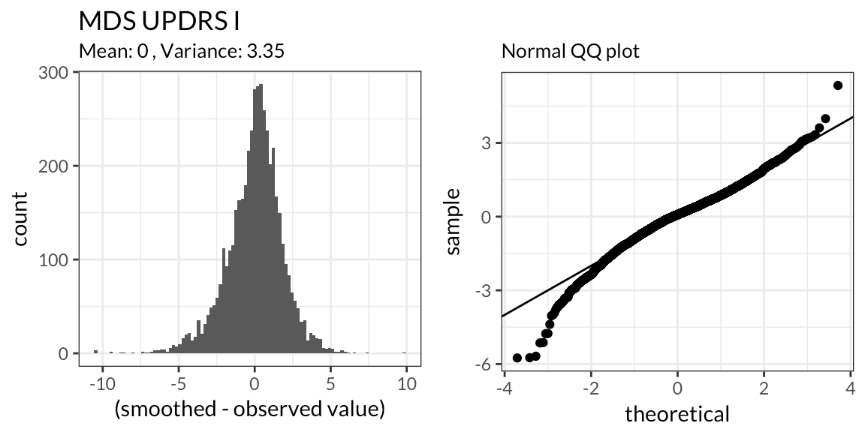


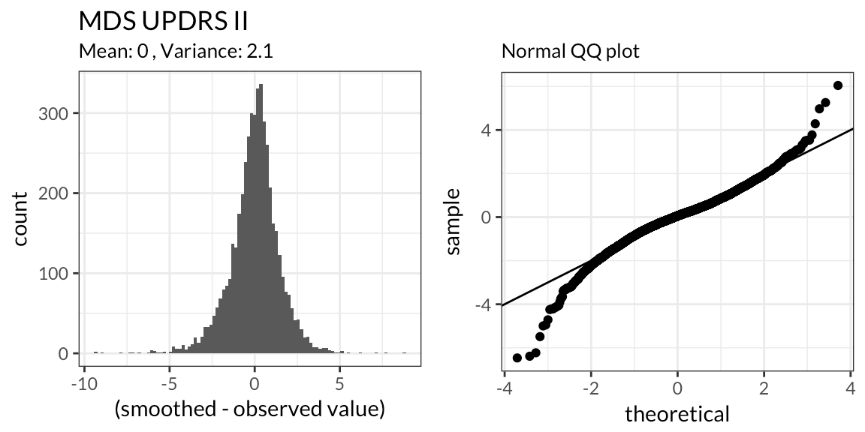


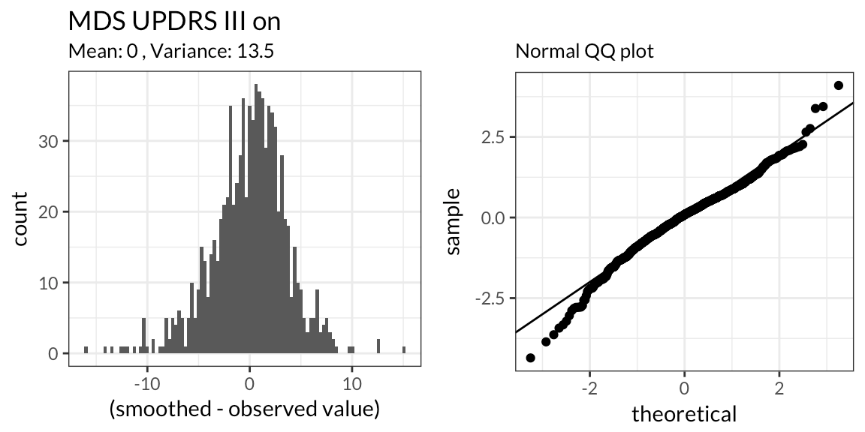


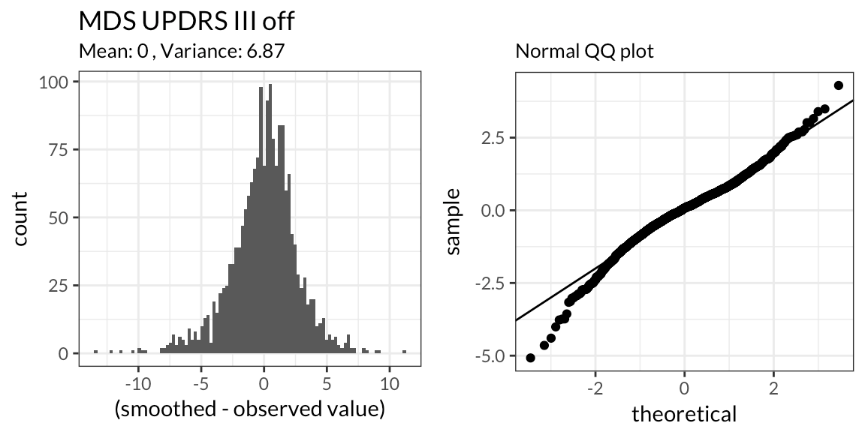


Figure 3: distribution of model residuals distribution i.e. the difference between the estimated true progression states and the observed values.

## F: Correlation between two subsequent change scores on generated data


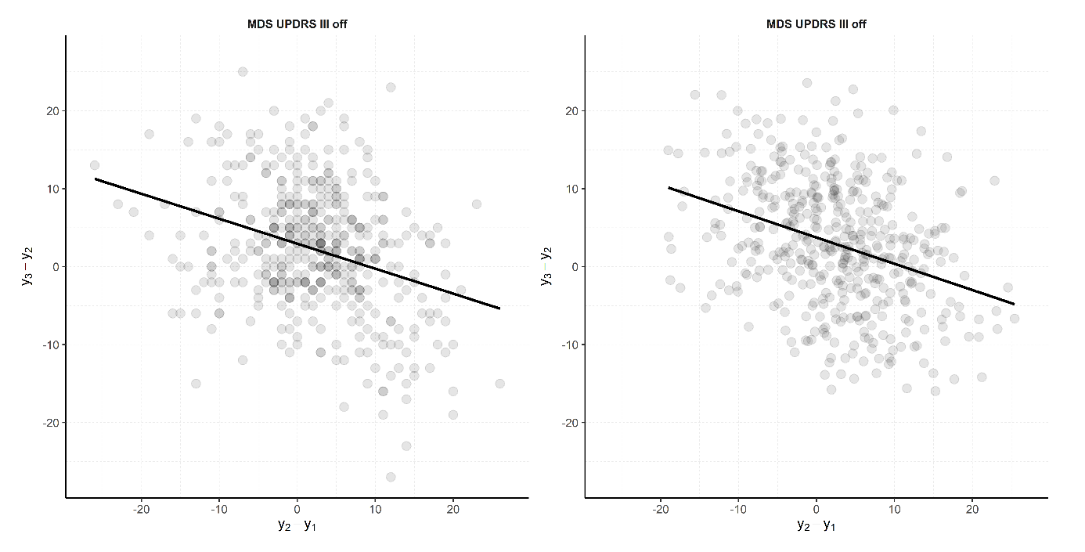


Figure 4: relation between two subsequent 1-year change scores of the MDS-UPDRS part III (OFF) on the PPMI dataset (left) and simulated data based on the estimated model (right). To create the plot of simulated data, we created 10 samples, each with the same amount of three consecutive yearly measurements as the original dataset. Each of these samples displayed a negative correlation between two subsequent change scores (one representative example is shown). Lines are fitted by linear regression on the response variable y3-y2.

## G: Mean and variance of change scores during study follow-up


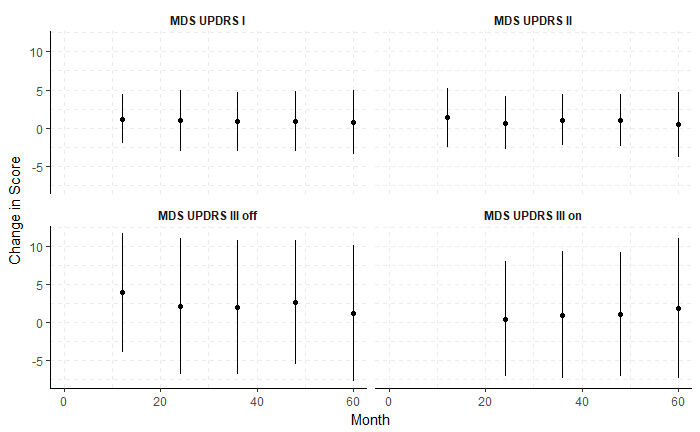


Figure 5: population mean change scores in year 1 to 5 for each subscale of the MDS-UPDRS (bars represent +/- one standard deviation).

Given the large overlap in the distribution of yearly changes scores (figure 5), we estimated one parameter for the average yearly progression for each part of the MDS-UPDRS. It would be possible to estimate separate parameters for each year, but we see two drawbacks of this. First, it would have increased the model’s complexity and therefore also the variance of the parameter estimates. Second, the interpretation of separate parameters for each year since the study start would not be straightforward; the time of diagnosis varies from 0 to 2 years from the study start and the diagnosis is not made at a specific point in all patients’ disease course, but varies because of patient’s and doctor’s delay. Assessing the scale’s behavior in the different disease stages is in our view the best compromise (where we focus on an early PD population).

## H: Relation between time since last medication intake and MDS-UPDRS scores


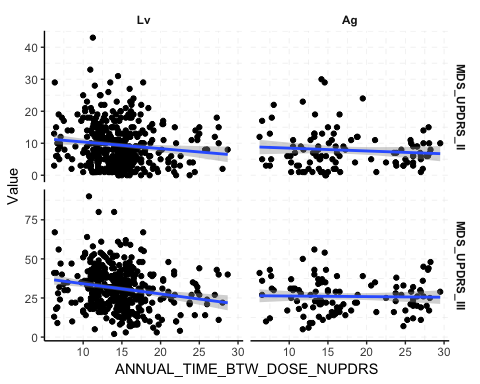


Figure 6: correlation between the time between the assessment and the last intake of dopaminergic medication for patients taking only levodopa (Lv, left column) and patients taking only dopamine agonists (Ag, right column), for part II and part III (off) of the MDS-UPDRS. Blue line is fitted by linear regression on the MDS-UPDRS score.

As mentioned in the results section, the estimated within-subject reliability of the MDS-UPDRS part III (off) was higher when selecting assessments with >14 hours since last medication intake in comparison with using a >6 hours threshold. Because the time since last medication intake was not randomized in the PPMI study, we should be cautious with inferring a causal relationship from these findings and consider the possibility of confounding (i.e. there might be a factor that impacts both the within-person reliability and the time since last intake). Figure 6 shows the relationship between the exact time since last intake and the observed MDS-UPDRS part III off scores. For the dopamine agonists, there is no obvious correlation. Surprisingly, when we consider levodopa we observe a negative correlation; patients with an increased time since last intake show lower (better) part III (off) scores. Because there is no plausible explanation for this to be a causal effect in the direction “increased time since last intake 🡪 lower part III scores” (it is well known that levodopa *improves* motor function as measured by part III), we suspect that this correlation might be explained by a causal effect in the other direction: patients who are worse in terms of motor symptoms may be more likely to have less time between last intake and assessment (e.g. because their regular medication schedule has a higher frequency, and/or they are less willing/able to withhold their medication for a longer period because they are more burdened by their off symptoms). This is supported by the similar negative correlation between the time since last intake and the part II score, in which the causal direction “increased time since intake 🡪 decreased part II scores” is even less likely because part II scores are not supposed to be affected at all by the time since last intake. Together, these findings indicate that we cannot rule out that the observed improvement in within-subject reliability is confounded by clinical characteristics. It also means that it is not straightforward to estimate the (causal) effect size of extending the time since last intake on part III off scores in this observational study. For this reason, we did not adjust the part III (off) scores for the effect of the exact time since last medication intake in the main analysis.
